# Sofosbuvir–velpatasvir plus ribavirin in Japanese patients with genotype 1 or 2 hepatitis C who failed direct-acting antivirals

**DOI:** 10.1007/s12072-018-9878-6

**Published:** 2018-07-20

**Authors:** Namiki Izumi, Tetsuo Takehara, Kazuaki Chayama, Hiroshi Yatsuhashi, Koichi Takaguchi, Tatsuya Ide, Masayuki Kurosaki, Yoshiyuki Ueno, Hidenori Toyoda, Satoru Kakizaki, Yasuhito Tanaka, Yoshiiku Kawakami, Hirayuki Enomoto, Fusao Ikeda, Deyuan Jiang, Shampa De-Oertel, Brian L. McNabb, Gregory Camus, Luisa M. Stamm, Diana M. Brainard, John G. McHutchison, Satoshi Mochida, Masashi Mizokami

**Affiliations:** 1 Musashino Red Cross Hospital, Tokyo, Japan; 2 Osaka University Hospital, Osaka, Japan; 3 Hiroshima University Hospital, Hiroshima, Japan; 4 Nagasaki Medical Center, Nagasaki, Japan; 5 Kagawa Prefectural Central Hospital, Kagawa, Japan; 6 Kurume University Hospital, Fukuoka, Japan; 7 Yamagata University Hospital, Yamagata, Japan; 8 Ogaki Municipal Hospital, Gifu, Japan; 9 Gunma University Hospital, Gunma, Japan; 10 Nagoya City University Hospital, Aichi, Japan; 11 Hyogo College of Medicine Hospital, Hyogo, Japan; 12 Okayama University Hospital, Okayama, Japan; 13 Gilead Sciences, Inc., Foster City, CA USA; 14 Saitama Medical University Hospital, Saitama, Japan; 15 National Center for Global Health and Medicine, Chiba, Japan

**Keywords:** DAA-experienced, NS5B polymerase inhibitor, NS5A inhibitor, Antiviral resistance, Salvage therapy

## Abstract

**Background/purpose:**

In Japan, there is a growing population of patients with chronic hepatitis C virus (HCV) infection who failed a direct-acting antiviral (DAA)-based regimen. In this Phase 3 study, we evaluated sofosbuvir–velpatasvir plus ribavirin in Japanese patients with genotype 1 or 2 HCV infection who previously received DAAs.

**Methods:**

Patients were randomized 1:1 to receive sofosbuvir–velpatasvir plus ribavirin for 12 or 24 weeks. Randomization was stratified by HCV genotype and presence of cirrhosis. The primary endpoint was sustained virologic response 12-week post-treatment (SVR12).

**Results:**

Of 117 participants, 81% had HCV genotype 1 infection, 33% had cirrhosis, and 95% had NS5A resistance-associated substitutions (RAS) at baseline. Overall, SVR12 rates were 97% (58/60; 95% CI 88–100%) with 24 weeks of treatment and 82% (47/57; 95% CI 70–91%) with 12 weeks. For HCV genotype 1 and 2 infected patients, the SVR12 rates with 24 weeks of treatment were 98% and 92%, respectively. In both treatment groups, SVR12 rates in HCV genotype 1 patients were statistically superior to a historical control rate of 50% (*p* < 0.001). For patients with NS5A RASs at baseline, 85% (46/54) in the 12-week group and 96% (54/56) in the 24-week group achieved SVR12. The most common adverse events were upper respiratory tract viral infection, anemia, and headache. Three (2.6%) patients discontinued treatment because of adverse events.

**Conclusion:**

Sofosbuvir–velpatasvir plus ribavirin was highly effective and well tolerated in Japanese patients who previously failed a DAA-based regimen. Baseline NS5A RASs did not affect treatment outcomes.

## Introduction

In Japan, there is a growing population of patients with chronic hepatitis C virus (HCV) infection who did not achieve sustained virologic response (SVR) with a direct-acting antiviral (DAA) regimen. The standard of care in Japan for chronic HCV infection has been evolving since the first DAA agent, telaprevir, was approved in 2011 for use in combination with peginterferon-alfa and ribavirin. In 2014, the all-oral regimen of daclatasvir, HCV NS5A inhibitor, and asunaprevir, HCV NS3/4A protease inhibitor, was approved for patients with chronic HCV genotype 1 infection [1]. Although the combination provided an interferon- and ribavirin-free treatment option, its overall efficacy has been suboptimal compared to newer DAA-based regimens. In a study of 222 Japanese patients with HCV genotype 1b, 15% experienced virologic failure with daclatasvir plus asunaprevir [2]. Failure rates were higher (59%) in patients with baseline NS5A resistance-associated substitutions (RASs), and treatment failure was associated with the emergence of RASs in the gene sequences for both NS5A and NS3/4. Separate analyses have evaluated the RAS profiles of patients who failed treatment with daclatasvir and asunaprevir. In one study, 63% of patients had dual NS5A RASs at L31 and Y93 at the time of failure [3]. A second study demonstrated that 91% had RASs at the time of virologic failure, including 52% with 2 RASs, 27% with 3 RASs, and 6% with deletions at NS5A sites 29 or 32 [4].

At the time this study was initiated, Japanese patients with HCV genotype 1 who had failed daclatasvir plus asunaprevir had very limited and complicated treatment options. The 2017 Japanese Society for Hepatology guidelines for hepatitis C treatment recommended that daclatasvir plus asunaprevir failures who were eligible to receive interferon be retreated with the NS3/4A inhibitor simeprevir plus peginterferon and ribavirin [5]. Those who were intolerant to or ineligible for interferon were recommended to receive ledipasvir–sofosbuvir as long as they did not have multiple resistance mutations in the NS5A region. For patients who did have multiple NS5A resistance mutations, who comprise the majority of daclatasvir plus asunaprevir failures [3, 4], a “wait-and-see” approach was recommended. Such patients had limited retreatment options, and they were typically excluded from clinical trials of novel HCV drugs.

The combination of sofosbuvir, NS5B polymerase inhibitor, with velpatasvir, NS5A inhibitor, is a once-daily, oral, pan-genotypic single-tablet regimen that is well tolerated and leads to high SVR rates (95–99%) in patients with or without compensated cirrhosis [6, 7]. Combining sofosbuvir–velpatasvir with ribavirin has the potential to be a salvage regimen for Japanese patients who have failed a DAA-containing regimen. In a previous Phase 2 study of patients who were DAA-experienced, treatment with sofosbuvir–velpatasvir plus ribavirin for 24 weeks resulted in SVR12 rates of 97% in patients with HCV genotype 1 and 93% in those with HCV genotype 2 [8]. In this Phase 3 study, we evaluated the efficacy and safety of sofosbuvir–velpatasvir plus ribavirin for 12 or 24 weeks in Japanese patients with genotype 1 HCV infection who were previously treated with NS5A inhibitor or genotype 2 HCV infection with any DAA-containing regimen.

## Methods

### Patients

Patients ≥ 20 years old with plasma HCV RNA ≥ 10^4^ IU/mL and chronic genotype 1 or 2 HCV infection that had previously not achieved SVR with a DAA-containing regimen lasting at least 4 weeks were eligible to enroll. For patients with HCV genotype 1, the DAA regimen must have included NS5A inhibitor. Patients without cirrhosis or with compensated cirrhosis were eligible for participation; the presence of cirrhosis was determined by either (1) liver biopsy with Metavir 4 or Ishak ≥ 5 scores; (2) Fibroscan > 12.5 kPa; or (3) FibroTest score ≥ 0.75. Key exclusion criteria included noncompliance with the most recent DAA-containing regimen, previous discontinuation of sofosbuvir and ribavirin because of intolerance, body weight < 40 kg, platelets < 50,000/µL, hemoglobin < 10 g/dL, alanine aminotransferase or aspartate aminotransferase > 10 × upper limit of normal (ULN); direct bilirubin > 1.5 × ULN; hemoglobin A1c > 8.5%; creatinine clearance (Cockcroft–Gault) < 50 mL/min; albumin < 3 g/dL; International Normalized Ratio of prothrombin time > 1.5 × ULN; infection with hepatitis B or HIV; or porphyria.

### Study design

This was a Phase 3, multicenter, open-label study. Via an interactive web response system, patients were randomly assigned 1:1 to 12 or 24 weeks of treatment with sofosbuvir–velpatasvir (400 mg/100 mg) fixed-dose combination tablet once-daily and weight-based ribavirin (REBETOL^®^, MSD KK) 600–1000 mg divided twice daily. Randomization was stratified by cirrhosis status (presence or absence) and HCV genotype (1 or 2). Approximately 90 patients with HCV genotype 1 and 20 patients with HCV genotype 2 were targeted for enrollment. Across the study population, approximately 20 were to have compensated cirrhosis. After completing 12 or 24 weeks of treatment, all patients underwent follow-up visits at post-treatment weeks 4, 12, and 24.

### Study oversight

The study protocol was approved by the review board or ethics committee of each institution prior to study initiation. The study was conducted in accordance with the International Conference on Harmonization Good Clinical Practice Guidelines and the Declaration of Helsinki. Patients provided written informed consent before undertaking any study-related procedures.

### Assessments

Screening assessments included measurement of plasma HCV RNA level, HCV genotyping, *IL28B* genotyping, and standard laboratory and clinical tests. HCV RNA levels were quantified using the COBAS Ampliprep/COBAS TaqMan HCV Test, v2.0 (Roche Molecular Systems, Inc., Branchburg, NJ), which has a lower limit of quantitation (LLOQ) of 15 IU/mL. HCV genotype and subtype was determined using the Siemens VERSANT^®^ HCV Genotype INNO-LiPA2.0 Assay. *IL28B* genotype was determined by polymerase chain reaction amplification of the single-nucleotide polymorphism rs12979860, with sequence-specific forward and reverse primers and allele-specific fluorescently labeled TaqMan^®^ minor groove binder probes.

Plasma HCV RNA levels were evaluated at screening; on day 1 of treatment; at treatment weeks 1, 2, 3, 4, 5, 6, 8, 10, and 12 for all patients and weeks 16, 20, and 24 for those receiving 24 weeks of treatment; and at post-treatment weeks 4, 12, and 24. Missing SVR values were imputed as a success if bracketed by values that were termed successes.

Plasma samples for viral sequencing were collected at all treatment and follow-up visits, following the same schedule as for HCV RNA evaluation. RASs present in more than 15% of the sequence reads are reported. Deep sequencing of the NS5A and NS5B coding regions was performed on samples obtained from all patients at baseline and from those with virologic failure at the time of failure.

Safety assessments included physical examinations and vital sign assessments conducted at all study visits. In addition, adverse events and concomitant medication intake were ascertained and clinical laboratory assessments were collected at screening, every treatment visit, and at the post-treatment week 4 visit.

### Endpoints

The primary efficacy endpoint was achievement of SVR12, defined as having HCV RNA < LLOQ 12 weeks after discontinuing study drugs. The primary safety endpoint was discontinuation of study drugs due to adverse events.

### Statistical analyses

Because of the limited number of patients with HCV genotype 2 patients in this study, the sample size justification was based on genotype 1 patients only. A sample size of 45 HCV genotype 1 patients in each treatment group was to provide over 90% power for the primary efficacy analysis, which was to detect at least 27% improvement in SVR12 rate from a historical control rate of 50% using a two-sided exact one-sample binomial test at significance level of 0.025 with Bonferroni alpha adjustment. The 50% SVR null rate was derived from SVR rates of 43% (59/137) and 59% (57/96) (116/233 = 50%) for treatment-naive patients with genotype 1 HCV infection and high viral loads treated with peginterferon and ribavirin for 48 weeks cited in the Japanese package inserts for REBETOL^®^ Capsules 200 mg (MSD, July 2015, 19th version) and COPEGUS^®^ Tablets 200 mg (Chugai Pharmaceuticals, July 2015, 6th version), respectively. No statistical hypothesis testing was performed for the groups of patients with HCV genotype 2. A point estimate with two-sided 95% exact confidence interval using the binomial distribution (Clopper–Pearson method) was constructed for the SVR12 rates in each treatment group. Also explored in post hoc analyses were factors associated with treatment failure. Exact logistic regressions were conducted using the relapse rate in 3 groups: all patients, patients infected with genotype 1 in both treatment groups combined, or patients treated for 12 weeks. Analysis variables were selected based on the size of the population and potential for impacting treatment success. The factors analyzed included sex, age group (< 65 or ≥ 65 years), absence or presence of cirrhosis, baseline HCV RNA (< 5 log10 IU/mL or ≥ 5 log10 IU/mL), number of RAVs (< 2 or ≥ 2), absence or presence of the NS5A RAVs L31 in combination withY93, adherence rate (< 80% or ≥ 80%), treatment duration (12 or 24 weeks), and RBV dosage as a continuous variable measured by number of tablets taken.

## Results

### Patient population

**Fig. 1:**
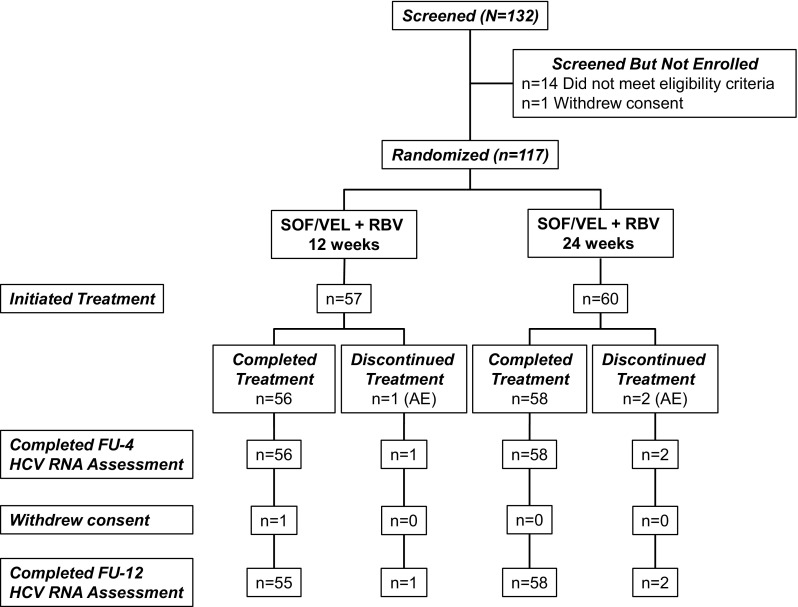
Patient disposition throughout the study. *FU-4* follow-up week 4, *FU-12*, follow-up week 12, *HCV* hepatitis C virus, *RBV* ribavirin, *SOF* sofosbuvir, *VEL* velpatasvir

From August of 2016 through March of 2017, 117 patients were treated at 18 study sites in Japan. The median age for the study population was 64 years (range 21–81) (Table 1). Thirty-three percent (39/117) of patients had cirrhosis. Fifty-seven percent (67/117) had a non-CC *IL*-*28B* genotype. Among patients with genotype 1 infection, 97% (92/95) had subtype 1b. Most patients (84%, 83/117) had undergone 2 or more prior DAA treatment regimens. The median (range) reported duration of the most recent prior DAA treatment was 14 (7–36) weeks in the 12-week group and 12 (6–36) in the 24-week group. Seventy-five percent (88/117) of patients were previously treated with both NS5A and NS3/4 inhibitors, including 8 patients who had also been treated with NS5B inhibitor. Among patients with genotype 1 HCV infection, the most common prior treatment regimen was daclatasvir plus asunaprevir (86%, 82/95), and, among patients with genotype 2 HCV infection, the most common prior DAA was sofosbuvir (91%, 20/22). Ninety-five percent of patients (110/116) had 1 or more NS5A RASs at baseline, including 71% (82/116) with 2 or more NS5A RASs. Of the 117 patients who were enrolled, 114 (97%) completed treatment (Fig. 1).

**Table 1:** Patient demographics and baseline characteristics

	Sofosbuvir-velpatasvir + ribavirin					
	Genotype 1		Genotype 2		Total	
	12 weeks (*n* = 47)	24 weeks (*n* = 48)	12 weeks (*n* = 10)	24 weeks (*n* = 12)	12 weeks (*n* = 57)	24 weeks (*n* = 60)
Mean (range) age, years	63 (38–81)	64 (35–79)	59 (21–76)	61 (46–70)	62 (21–81)	63 (35–79)
Female, *n* (%)	29 (62)	28 (58)	5 (50)	5 (42)	34 (60)	33 (55)
Race, *n* (%)						
Asian	47 (100)	48 (100)	10 (100)	12 (100)	57 (100)	60 (100)
Median (range) BMI, kg/m^2^	24 (18–33)	23 (18–30)	23 (21–29)	24 (18–36)	24 (18–33)	23 (18–36)
Genotype, *n* (%)						
1	47 (100)	48 (100)	–	–	47 (82)	48 (80)
1a	2 (4)	1 (2)	–	–	2 (4)	1 (2)
1b	45 (96)	47 (98)	–	–	45 (79)	47 (78)
2	–	–	10 (100)	12 (100)	10 (18)	12 (20)
2a	–	–	7 (70)	8 (67)	7 (12)	8 (13)
2b	–	–	3 (30)	4 (33)	3 (5)	4 (7)
Mean (SD) HCV RNA, log_10_ IU/mL	6.2 (0.47)	6.2 (0.51)	6.6 (0.46)	6.2 (0.86)	6.3 (0.49)	6.2 (0.58)
HCV RNA ≥ 800,000 IU/mL, *n* (%)	37 (79)	38 (79)	9 (90)	8 (67)	46 (81)	46 (77)
No. of prior DAAs, *n* (%)						
1	2 (4)	0	9 (90)	8 (67)	11 (19)	8 (13)
2	34 (72)	39 (81)	1 (10)	2 (17)	35 (61)	41 (68)
≥ 3	11 (23)	9 (19)	–	2 (17)	11 (19)	11 (18)
No. of prior treatment regimens, *n* (%)						
1	13 (28)	13 (27)	2 (20)	6 (50)	15 (26)	19 (32)
2	15 (32)	18 (38)	5 (50)	3 (25)	20 (35)	21 (35)
3	8 (17)	5 (10)	2 (20)	2 (17)	10 (18)	7 (12)
≥ 4	11 (23)	12 (25)	1 (10)	1 (8)	12 (21)	13 (22)
Cirrhosis, *n* (%)						
Yes	16 (34)	18 (38)	2 (20)	3 (25)	18 (32)	21 (35)
No	31 (66)	30 (63)	8 (80)	9 (75)	39 (68)	39 (65)
Prior DAAs by class, *n* (%)						
NS5A + NS3 ± NS5B	44 (94)	41 (85)	1 (10)	2 (17)	45 (79)	43 (72)
NS5B ± NS3	–	–	9 (90)	9 (75)	9 (16)	9 (15)
NS5A ± NS5B	3 (6)	7 (15)	–	1 (8)	3 (5)	8 (13)
Prior DAAs, *n* (%)						
DCV	44 (94)	40 (83)	–	1 (8)	44 (77)	41 (68)
DCV + ASV	42 (89)	40 (83)	–	1 (8)	42 (74)	41 (68)
SOF	3 (6)	11 (23)	9 (90)	11 (92)	12 (21)	22 (37)
LDV-SOF	3 (6)	11 (23)	–	1 (8)	3 (5)	12 (20)
DCV + ASV and LDV–SOF	1 (2)	4 (8)	–	–	1 (2)	4 (7)
SMV					6	7
TVR					2	1
VAN					–	1
GRZ + ELB					1	1
OMB + PAR					1	1
GLE + PIB					1	–
*IL*-*28B*, *n* (%)						
CC	15 (32)	21 (44)	8 (80)	6 (50)	23 (40)	27 (45)
CT	28 (60)	20 (42)	1 (10)	6 (50)	29 (51)	26 (43)
TT	4 (9)	7 (15)	1 (10)	–	5 (9)	7 (12)
NS5A resistance-associated substitutions, *n*/*n* (%)						
Without	1/46 (2)	2/48 (4)	1/10 (10)	2/12 (17)	2/56 (4)	4/60 (7)
With	45/46 (98)	46/48 (96)	9/10 (90)	10/12 (83)	54/56 (96)	56/60 (93)
1	5/46 (11)	6/48 (13)	9/10 (90)	8/12 (67)	14/56 (25)	14/60 (23)
≥2	40/46 (87)	40/48 (83)	–	2/12 (17)	40/56 (71)	42/60 (70)
Y93 any ± other	41/46 (89)	39/48 (81)	–	–	41/56 (73)	39/60 (65)
L31 any ± other	38/46 (83)	42/48 (88)	9/10 (90)	10/12 (83)	47/56 (84)	52/60 (87)
P32 deletion ± other	2/46 (4)	3/48 (6)	–	–	2/56 (4)	3/60 (5)

*ASV* asunaprevir, *BMI* body mass index, *DAA* direct-acting antiviral, *DCV* daclatasvir, *ELB* elbasvir, *GLE* glecaprevir, *GT* genotype, GRZ grazoprevir, *HCV* hepatitis C virus, *LDV* ledipasvir, *OMB* ombitasvir, *PAR* paritaprevir, *PIB* pibrentasvir, *SMV* simeprevir, *SOF* sofosbuvir, *TVR* telaprevir, *VAN* vaniprevir

### Efficacy

Overall, SVR12 rates were higher with 24 weeks versus 12 weeks of treatment (Table 2). In the 12- and 24-week treatment groups, 82% (47/57; 95% CI 70–91%) and 97% (58/60; 95% CI 88–100%) of patients achieved SVR12, respectively. Among patients with HCV genotype 1, SVR12 rates were 85% (40/47; 95% CI 72–94%) with 12 weeks and 98% (47/48; 95% CI 89–100%) with 24 weeks. The SVR12 rates of sofosbuvir–velpatasvir plus ribavirin for 12 weeks (*p* < 0.001) and 24 weeks (*p* < 0.001) in HCV genotype 1 patients were both statistically superior to the historical control rate of 50%. For patients with HCV genotype 2, SVR12 rates were 70% (7/10; 95% CI 35–93%) for 12 weeks and 92% (11/12; 95% CI 62–100%) for 24 weeks. Comparatively, the difference in SVR12 rate for the treatment groups overall was statistically significant (24 weeks compared with 12 weeks for all patients, *p* = 0.023); however, the differences in the SVR12 rates by genotype for the treatment groups were not statistically significant (for patients with genotype 1, *p* = 0.0548; for patients with genotype 2, *p* = 0.4511).

**Table 2:** Treatment response to sofosbuvir-velpatasvir + ribavirin

	Sofosbuvir-velpatasvir + ribavirin					
	Genotype 1		Genotype 2		Total	
	12 weeks (*n* = 47)	24 weeks (*n* = 48)	12 weeks (*n* = 10)	24 weeks (*n* = 12)	12 weeks (*n* = 57)	24 weeks (*n* = 60)
HCV RNA < 15 IU/mL, *n*/*n* (%)						
On treatment						
Week 1	12/47 (26)	11/48 (23)	0/10	4/12 (33)	12/57 (21)	15/60 (25)
Week 2	29/46 (63)	34/48 (71)	7/10 (70)	8/12 (67)	36/56 (64)	42/60 (70)
Week 4	45/46 (98)	47/48 (98)	10/10 (100)	12/12 (100)	55/56 (98)	59/60 (98)
Week 8	46/46 (100)	48/48 (100)	10/10 (100)	12/12 (100)	56/56 (100)	60/60 (100)
Week 12	46/46 (100)	47/47 (100)	10/10 (100)	12/12 (100)	56/56 (100)	59/59 (100)
Week 16	–	46/46 (100)	–	12/12 (100)	–	58/58 (100)
Week 24	–	46/46 (100)	–	12/12 (100)	–	58/58 (100)
After treatment						
Week 4	42/47 (89)	47/48 (98)	7/10 (70)	12/12 (100%)	49/57 (86)	59/60 (98%)
Week 12 (SVR12)	40/47 (85)	47/48 (98)	7/10 (70)	11/12 (92%)	47/57 (82)	58/60 (97%)
95% CI	72–94%	89–100%	35–93%	62–100%	70–91%	89–100%
Week 24 (SVR24)	40/47 (85)	47/48 (98)	7/10 (70)	11/12 (92%)	47/57 (82)	58/60 (97%)
95% CI	72–94%	89–100%	35–93%	62–100%	70–91%	89–100%
Virologic failure, *n* (%)						
On treatment	0	0	0	0	0	0
Relapse	6	1	3	1	9	2
Completed treatment	6	1	3	1	9	2
Discontinued treatment	0	0	0	0	0	0
Other virologic outcome, *n* (%)						
Did not complete treatment	1^a^	0	0	0	1	0

*GT* genotype, *HCV* hepatitis C virus, *SVR12* sustained virologic response 12 weeks after treatment

^a^Patient terminated participation on day 4 of treatment because of an adverse event (rash)

Results were similar between patients with and without cirrhosis in both treatment groups (Table 3). In the 12 week group, SVR12 rates were 82% (32/39) for those without cirrhosis and 83% (15/18) for those with compensated cirrhosis. In the 24-week group, they were 95% (37/39) in patients without cirrhosis and 100% (21/21) in those with cirrhosis.

**Table 3:** SVR12 by cirrhosis, prior direct-acting antivirals, and baseline resistance-associated substitutions

	Sofosbuvir-velpatasvir + ribavirin					
	Genotype 1		Genotype 2		Total	
	12 weeks	24 weeks	12 weeks	24 weeks	12 weeks weeks	24 weeks
Cirrhosis						
Yes	81% (13/16)	100% (18/18)	100% (2/2)	100% (3/3)	83% (15/18)	100% (21/21)
No	87% (27/31)	97% (29/30)	63% (5/8)	89% (8/9)	82% (32/39)	95% (37/39)
Prior DAAs by class						
NS5A + NS3 ± NS5B	86% (38/44)	98% (40/41)	100% (1/1)	100% (2/2)	87% (39/45)	98% (42/43)
NS5B ± NS3	–	–	67% (6/9)	89% (8/9)	67% (6/9)	89% (8/9)
NS5A ± NS5B	67% (2/3)	100% (7/7)	–	100% (1/1)	67% (2/3)	100% (8/8)
Prior DAAs						
DCV	84% (37/44)	98% (39/40)	–	100% (1/1)	84% (37/44)	98% (40/41)
DCV + ASV	86% (36/42)	98% (39/40)	–	100% (1/1)	86% (36/42)	98% (40/41)
SOF	100% (3/3)	100% (11/11)	67% (6/9)	91% (10/11)	75% (9/12)	96% (21/22)
LDV/SOF	100% (3/3)	100% (11/11)	–	100% (1/1)	100% (3/3)	100% (12/12)
DCV + ASV and LDV/SOF	100% (1/1)	100% (4/4)	–	–	100% (1/1)	100% (4/4)
NS3-containing regimens	50% (4/8)	100% (8/8)	–	0% (0/1)	50% (4/8)	89% (8/9)
Other DAA combinations	100% (2/2)	100% (1/1)	100% (1/1)	100% (1/1)	100% (3/3)	100% (2/2)
NS5A resistance-associated substitutions						
Without	100% (1/1)	100% (2/2)	0% (0/1)	100% (2/2)	50% (1/2)	100% (4/4)
With	87% (39/45)	98% (45/46)	78% (7/9)	90% (9/10)	85% (46/54)	96% (54/56)
1	100% (8/8)	100% (6/6)	78% (7/9)	88% (7/8)	86% (12/14)	93% (13/14)
≥ 2	85% (34/40)	98% (39/40)	–	100% (2/2)	85% (34/40)	98% (41/42)
Y93any ± other	85% (35/41)	100% (39/39)	–	–	85% (35/41)	100% (39/39)
L31any ± other	84% (32/38)	98% (41/42)	78% (7/9)	90% (9/10)	83% (39/47)	96% (50/52)
P32 deletion ± other	100% (2/2)	67% (2/3)	–	–	100% (2/2)	67% (2/3)

*ASV* asunaprevir, *DAA* direct-acting antiviral, *DCV* daclatasvir, *GT* genotype, *LDV* ledipasvir, *SOF* sofosbuvir

The SVR12 rates for patients with genotype 1 HCV infection previously treated with both NS5A and NS3/4 inhibitors, including those who had also used NS5B inhibitor, were 86% (38/44) and 98% (40/41) in the 12- and 24-week groups, respectively. SVR12 rates were 86% (36/42) and 98% (39/40) with 12 and 24 weeks of treatment, respectively, in patients previously treated with daclatasvir plus asunaprevir, 100% (3/3) and 100% (11/11) in those previously treated with ledipasvir–sofosbuvir, and 100% (1/1) and 100% (4/4) in patients previously treated with daclatasvir plus asunaprevir and then ledipasvir–sofosbuvir. The SVR12 rates in the 12- and 24-week groups for patients with genotype 2 HCV infection previously treated with sofosbuvir were 67% (6/9) and 91% (10/11), respectively.

No patients had virologic nonresponse. A total of 11 patients relapsed, 9 of whom were in the 12-week grosup. One patient terminated treatment on day 8 because of an adverse event and did not achieve SVR12. In post hoc logistic regression analyses of relapse in the overall population (n = 116), the only factor that was statistically significant was treatment duration, where the likelihood for relapse was 5.5-fold higher with 12 weeks than with 24 weeks (*p* = 0.0399). For genotype 1 patients in both treatment groups (n = 95) and in the 12 week group alone (n = 47), no factor was statistically significant.

### Viral resistance analyses

Among the 116 patients included in the resistance analysis population, the prevalence of baseline NS5A RASs was high and similar between the two treatment groups irrespective of genotype. Overall, 96% (54/56) in the 12-week group and 93% (56/60) in the 24-week group had baseline NS5A RASs. Most patients with genotype 1 HCV had 2 or more NS5A RASs (overall 85%, 80/94), including Y93 alone or in combination with other substitutions (overall 85%, 80/94) and P32 deletions (overall 5%, 5/94). The majority of those with a Y93 RAS also had L31 RAS (overall 89%, 71/80). Eighty-six percent (71/80) of patients with genotype 2 infection had 1 or 2 NS5A RASs at baseline (overall 86%, 19/22; genotype 2a 87%, 13/15; genotype 2b 86%, 6/7). All patients with genotype 2 infection and NS5A RAVs had L31M.

SVR12 was achieved in 85% (46/54) and 96% (54/56) of patients with baseline NS5A RASs in the 12- and 24-week groups, respectively (Table 3). Among those with two or more baseline NS5A RASs, 85% (34/40) in the 12-week group and 98% (41/42) in the 24-week group achieved SVR12. For patients with HCV genotype 1, SVR12 was achieved in 85% (35/41) and 100% (39/39) of those with any Y93 RAS, 82% (28/34) and 100% (37/37) for those with Y93 combined with L31 RASs, and 100% (2/2) and 67% (2/3) in patients with P32 deletions, in the 12- and 24-week groups, respectively. Among patients with genotype 2 infection with L31M RASs, 78% (7/9) and 90% (9/10) achieved SVR12 in the 12- and 24-week groups, respectively.

Seven patients (*n* = 4 HCV genotype 1b infection, *n* = 3 HCV genotype 2b infection) had NS5B RASs at baseline (*n* = 3 in the 12-week group and *n* = 4 in the 24-week group). All achieved SVR12.

None of the 11 patients who relapsed across the treatment groups developed treatment-emergent RASs at a cutoff of 15% or 1%.

### Safety

Eighty-one percent (46/57) of patients in the 12-week group and 75% (45/60) of patients in the 24-week group experienced an adverse event (Table 4). The most commonly reported adverse events were viral upper respiratory tract infection (28%), anemia (23%), and headache (11%). Anemia was reported at similar percentages in the 12- and 24-week treatment groups, 25% and 22%, respectively. Four patients, all in the 24-week group, experienced a Grade 3, serious adverse event; 2 had hepatocellular carcinoma, 1 had hepatic angiosarcoma, and 1 had pneumonia. None of the serious adverse events was considered related to study treatment.

**Table 4:** Adverse events and laboratory abnormalities

	Sofosbuvir–velpatasvir + ribavirin	
	12 weeks (*n* = 57)	24 weeks (*n* = 60)
No. (%) of patients with any adverse event	46 (81)	45 (75)
No. (%) of Grade 3 or 4 adverse events	0	4 (7)
No. (%) of patients with a serious adverse event	0	4 (7)
Adverse events leading to discontinuation of all study drug, *n* (%)	1 (2)	2 (3)
Deaths, *n*	0	0
Adverse events in ≥ 5% of patients in either treatment group, *n* (%)		
Upper respiratory tract viral infection	20 (35)	13 (22)
Anemia	14 (25)	13 (22)
Headache	11 (19)	2 (3)
Stomatitis	5 (9)	3 (5)
Eczema	4 (7)	2 (3)
Nausea	5 (9)	1 (2)
Pharyngitis	3 (5)	3 (5)
Pruritus	2 (4)	4 (7)
Back pain	4 (7)	1 (2)
Rash	2 (4)	3 (5)
Dry skin	0	4 (7)
Gastroenteritis	0	4 (7)
Malaise	1 (2)	3 (5)
Upper abdominal pain	3 (5)	0
Oral herpes	0	3 (5)
Upper respiratory tract inflammation	0	3 (5)
Serious adverse events, *n* (%)		
Hepatocellular carcinoma	0	2 (3)
Hepatic angiosarcoma	0	1 (2)
Pneumonia	0	1 (2)
Laboratory abnormalities (Grade 3 or above), *n* (%)		
Hyperglycemia, > 250 to 500 mg/dL	3 (5)	5 (8)
Lymphocytes, 350 to < 500/mm^3^	1 (2)	7 (12)
Hemoglobin, 7.0 to < 9.0 g/dL or decrease ≥ 4.5 g/dL	2 (4)	4 (7)
Hyponatremia, 121 to < 125 mmol/L	0	1 (2)
Neutrophils, 500 to < 750/mm^3^	0	1 (2)
Platelets, 25,000 to < 50,000/mm^3^	0	1 (2)
White blood cells, 1000–1500/mm^3^	0	1 (2)

Three patients had adverse events leading to premature discontinuation of treatment. One of them, in the 12-week group, discontinued on treatment day 8 because of rash and did not achieve SVR12. The rash was considered related to study treatment and resolved within 1 month. Another patient, in the 24-week group, had hepatic angiosarcoma that was considered unrelated to study treatment. This patient discontinued study drugs on day 97 of treatment and achieved SVR12. The third patient, also in the 24-week group, experienced moderately severe depression that was considered related to study treatment; the patient’s medical history was notable for a prior episode of depression related to treatment with peginterferon plus ribavirin. This patient discontinued after 5 weeks of treatment and achieved SVR12.

Ten patients had adverse events that led to ribavirin dose reduction (*n* = 9) or interruption (*n* = 1). All ten patients had anemia that was considered related to study treatment, and one also had headache considered related to study treatment. Seven of the ten reached SVR12; three experienced relapse. All three had genotype 2 HCV and were in the 12-week group.

No patients had Grade 4 laboratory abnormalities. The only Grade 3 laboratory abnormalities that occurred in more than one patient were hyperglycemia (*n* = 8), lymphocyte reduction (*n* = 8), and decreased hemoglobin levels (*n* = 6). All eight patients with Grade 3 hyperglycemia had a history of diabetes.

## Discussion

In this Phase 3 study in Japan, sofosbuvir–velpatasvir plus ribavirin was highly effective and well tolerated in patients with HCV genotype 1 or 2 infection with or without compensated cirrhosis who had not achieved sustained virologic response after the previous treatment with DAA-containing regimens, including NS5A inhibitors. In this study, extending duration of therapy with sofosbuvir–velpatasvir plus ribavirin to 24 versus 12 weeks resulted in higher SVR rates, and the difference was statistically significant. In a univariate regression analysis of all enrolled patients, the only factor significantly associated with relapse was shorter treatment duration, suggesting that 24 weeks of treatment is of benefit for all DAA-experienced patients. The results with 24 weeks of treatment in the current study are similar to a smaller, prior study of 24 weeks of sofosbuvir–velpatasvir plus ribavirin in DAA-experienced patients, which resulted in SVR12 rates of 97% in patients with HCV genotype 1 and 93% in those with HCV genotype 2 [8]. However, only 7% of patients in the prior study were infected with HCV genotype 1b, compared with 78% in the current study, and only 14% had at least 1 NS5A RASs at baseline, compared with 92% of the HCV genotype 1 patients in the current study.

The adverse event profile in this study was generally similar to those reported in the previous studies of regimens including sofosbuvir and ribavirin [9–12]. Three patients (2.6%) discontinued treatment because of an adverse event, yet despite the early discontinuation, 2 of them achieved SVR12. Typical with ribavirin-containing regimens, anemia occurred in approximately one-fifth of patients but did not result in treatment discontinuation in any patients.

The current Japanese treatment guidelines recommend glecaprevir-pibrentasvir as the first-line retreatment option for patients who have failed NS3/4A protease inhibitor and NS5A inhibitor, and who do not have baseline NS3/4 or NS5A RASs. The Phase 3 CERTAIN-1 study evaluated treatment with glecaprevir-pibrentasvir for 12 weeks in Japanese patients [13]. Of the 33 DAA-experienced subjects, 30 had previously been treated with daclatasvir and asunaprevir, 2 with peginterferon and ribavirin and simeprevir, and 1 with sofosbuvir and ribavirin. SVR12 was achieved by 94% (31/33) of patients, and both patients with virologic failure had genotype 1b HCV infection and P32 deletions in the NS5A region at baseline. One of the two patients with virologic failure also had the NS3 RAS D168V at baseline and emergent A156D/V at failure. In the United States, glecaprevir-pibrentasvir is not recommended for HCV genotype 1 patients who previously received both NS5A and NS3/4A inhibitors, and instead sofosbuvir–velapatasvir–voxilaprevir is recommended [14]. One clear benefit of sofosbuvir–velpatasvir plus ribavirin is that it can be used in patients with decompensated cirrhosis.

The previous studies have shown that patients with genotype 1b infection who were unsuccessfully treated with daclatasvir plus asunaprevir frequently have complex RAS profiles [3, 4]. Similar observations were made in this study, as the majority of genotype 1 patients had 2 or more NS5A RASs at baseline. Specific NS5A RASs associated with daclatasvir plus asunaprevir treatment failures that confer high levels of resistance to NS5A inhibitors include dual mutations at Y93 and L31 as well as P32 deletions. The dual NS5A RASs and P32 deletions have been associated with relapse in ledipasvir–sofosbuvir and glecaprevir-pibrentasvir re-treatment studies [13, 15–17]. In this study, the overall presence of NS5A substitutions or the presence of specific NS5A substitutions at baseline had no discernible effect on the rates of SVR12 with sofosbuvir–velpatasvir plus ribavirin. All 37 patients in the 24-week group with baseline Y93 and L31 RASs achieved SVR12. Furthermore, 4 of the 5 patients enrolled in the current study with a P32 deletion at baseline achieved SVR with 12 or 24 weeks of treatment.

The majority of patients in the current study with genotype 2a (87% [13/15]) or genotype 2b (86% [6/7]) had 1 or more NS5A RASs at baseline, all with L31M. In contrast, it was previously reported that worldwide 97% of patients with HCV genotype 2a and 39% of patients with HCV genotype 2b had L31M [18]. Our data suggest that there may be a higher prevalence of L31M in HCV genotype 2b strains circulating in Japan relative to the global population, although this is based on a small number of patients.

Prior studies have suggested that there is an association between the duration of prior DAA treatment and success in retreatment, with patients treated with shorter durations of all-oral NS5A inhibitor-based DAA therapy (4–8 weeks) having higher retreatment SVR rates compared to those initially treated for longer durations (10–12 weeks) [19, 20], a phenomenon, perhaps, resulting from greater virologic resistance developing during longer treatment. In the current study, the median duration of most recent prior DAA treatment was 12–14 weeks, and 95% of patients had baseline NS5A RASs. The high SVR12 (97%) rate in among patients who received 24 weeks of treatment demonstrates that the inclusion of ribavirin and the extended treatment duration are effective in treating this highly treatment-experienced patient population infected with resistant HCV.

This study was designed to evaluate two durations of treatment with the same regimen of sofosbuvir–velpatasvir and ribavirin. It did not include a ribavirin-free arm, because the population consisted of DAA-experienced patients expected to have complex resistance profiles who would benefit from ribavirin in addition to two highly potent direct-acting antivirals. As such, the study does not give insight into whether the addition of ribavirin could be unnecessary for some patients. The sample size precludes meaningful analyses of subgroups of patients.

Further limitations of this study are the small number of patients with genotype 1a or genotype 2 HCV infections. The distribution of genotypes and subtypes is representative of the HCV population in Japan, which is predominantly genotype 1b [21]. The small sample size of HCV genotype 2 patients makes it difficult to interpret the high rate of relapse with 12 weeks of treatment, which does not seem to be attributable to the presence of L31M RASs nor the presence of cirrhosis. The three patients with genotype 1a infection were all successfully treated in the current study; however, the sample size is too small to predict treatment outcomes in a larger population with this subtype. Another limitation of the study is that there were few patients who had previously been treated with other next-generation DAA regimens, such as glecaprevir–pibrentasvir (*n* = 1), elbasvir–grazoprevir (*n* = 2), and ritonavir-boosted ombitasvir–pariteprevir (*n* = 2); all of these patients were successfully treated (data not shown).

In summary, sofosbuvir–velpatasvir plus ribavirin for 24 weeks was highly effective and well tolerated in Japanese patients with chronic HCV genotype 1 or 2 infection who previously failed treatment with a DAA. The presence of NS5A or NS5B RASs at baseline, including those associated with virologic failure with other DAA regimens, did not impact treatment outcomes. Sofosbuvir–velpatasvir plus ribavirin for 24 weeks is an effective salvage regimen for this population with limited treatment options.

## Abbreviations

ASV: Asunaprevir
BMI: Body mass index
DAA: Direct-acting antiviral
DCV: Daclatasvir
ELB: Elbasvir
GLE: Glecaprevir
GRZ: Grazoprevir
GT: Genotype
HCV: Hepatitis C virus
LDV: Ledipasvir
LLOQ: Lower limit of quantification
OMB: Ombitasvir
PAR: Paritaprevir
PIB: Pibrentasvir
RAS: Resistance-associated substitution
RBV: Ribavirin
SMV: Simeprevir
SOF: Sofosbuvir
SVR: Sustained virological response
TVR: Telaprevir
ULN: Upper limit of normal
VAN: Vaniprevir
VEL: Velpatasvir
